# Supplementary material for: Cell fate potentials and switching kinetics uncovered in a classic bistable genetic switch
Source: Nat Commun. 2018 Jul 17;9:2787. doi: 10.1038/s41467-018-05071-1 (PMC6050291; doi:10.1038/s41467-018-05071-1)
Supplement: Supplementary file 1 — Supplementary Information [file 41467_2018_5071_MOESM1_ESM.pdf]

Supplementary Information for

New Cell Fate Potentials and Switching Kinetics Uncovered in a Classic Bistable Genetic Switch

Xiaona Fang[1,2,3] , Qiong Liu[1], Christopher Bohrer[2], Zach Hensel[2,5], Wei Han[3], Jin Wang[1,3,4,]*, Jie Xiao[2,]*

[1]State Key Laboratory of Electroanalytical Chemistry, Changchun Institute of Applied Chemistry, Changchun, China.

[2]Department of Biophysics and Biophysical Chemistry, Johns Hopkins School of Medicine, Baltimore, Maryland, USA.

[3]College of Physics, Jilin University, Changchun, China.

[4]Department of Chemistry and Physics, Stony Brook University, Stony Brook, New York, USA

[5]Current address: Instituto de Tecnologia Química e Biológica António Xavier, Universidade Nova de Lisboa, Av. da República, 2780-157 Oeiras, Portugal

*Correspondence and requests for materials should be addressed to J.W. (jin.wang.1@stonybrook.edu) and J.X. (xiao@jhmi.edu).

Supplementary Tables

Supplementary Table 1: Strains, plasmids and primers used in the study.

Strain	Genotype	Source
XF103	*BW25113 (∆lacI)::lacI-venus-ub-cI/Cro*	This work
ZH051	*BW25113 (∆lacI)::tsr-venus-ub-cI/Cro*	This work
XF001	*MG1655 (∆lacI)::LP1-tet^R-LP2*	This work
XF002	*ZH051(pZZ6/pCG001)*	This work
XF003	*XF103(pZZ6/pCG001)*	This work
XF004	*ZH051(pZZ6/pXF011)*	This work
XF204	*MG1655(∆lacI)::tsr-venus-ub-cI^{857}/lacI-venus-ub-cro*	This work
XF214	*XF204* with GTG start codon for *lacI*	This work
XF224	*XF204* with mutated P_R (-32 A to G)[1]	This work
XF016	*MG1655(∆lacI)::tsr-venus-ub-cI^{857}/Cro (pZZ6/pCG001)*	This work
XF206	*MG1655(∆lacI)::tsr-venus**(G65A and G67A)-*ub-cI^{857}/lacI-venus-ub-cro (pZZ6/pCG001)*	This work
XF225	*XF016* with mutated P_R (-32 A to G)[1]	This work
XF226	*XF206* with mutated P_R (-32 A to G)[1]	This work

Plasmid	Description	Source
pTKIP	Donor plasmid	Ref[2]
pTKRED	Helper plasmid	Ref[2]
pTKS/CS	Landing pad vector	Ref[2]
pVS143	PCR template to generate lacI-Venus	Ref[3]
pCG001	Plasmid carry Ubp1	Ref[4]
pZZ6	RK2 plasmid carry lacO^{256}	Ref[5]
pZH051	*tsr-venus-ub-cI/cro* on pBR322 vector	Ref[6]
pZH016	*tsr-venus-ub-cI^{857}/cro* on pBR322 vector	Ref[6]
pXF103	*lacI-venus-ub-cI/cro* on pBR322 vector	This work
pXF104	*tsr-venus-ub-cI^{857}/lacI-venus-ub-cro* on pBR322 vector	This work
pXF204	*tsr-venus-ub-cI^{857}/lacI-venus-ub-cro* on pTKIP vector	This work
pXF016	*tsr-venus-ub-cI^{857}/cro* on pTKIP vector	This work
pXF106	*tsr-venus**(G65A and G67A)-*ub-cI^{857}/cro* on pBR322 vector	This work
pXF206	*tsr-venus**(G65A and G67A)-*ub-cI^{857}/lacI-venus-ub-cro* on pTKIP	This work
pXF214	*tsr-venus-ub-cI^{857}/lacI^{GTG}-venus-ub-cro* on pTKIP vector	This work
pXF224	*tsr-venus-ub-cI^{857}/lacI-venus-ub-cro* on pTKIP vector with P_R (-32 A to G)	This work
pXF225	*tsr-venus-ub-cI^{857}/cro* on pTKIP vector with P_R (-32 A to G)	This work
pXF226	*tsr-venus**(G65A and G67A)-*ub-cI^{857}/lacI-venus-ub-cro* on pTKIP with P_R (-32 A to G)	This work
pZH102	PCR template to generate *lacI-mCherry*	Ref[7]
pXF004	pCG001 with *lacI-mCherry* under promoter pBAD	This work

pXF011	pCG001 with *lacI-mCherry* under promoter BBa_J23103[8]	This work

Primer	Sequence
P1	TCAACTCGAGACCGCACTTGTACAGCTCGTCCATGC
P2	GCCGCTCGAGGAAGGAGCCCTTCACCATG
P3	CGCGGTATGGCATGATAGCGCCCGGAAGAGAGTCAATTCAGGGTGGTGAATCTATCGACTACGCGATCATGG
P4	CAATCAACTGTTTACCTTGTGGAGCGACATCCAGAGGCACTTCACCGCTTGCGATCTTCCCCATCGGTGATG
P5	ATGGAACAACGCATAACCCTG
P6	ACAACCTCCTTAGTACATGCAACC
P7	ATGAAACCAGTAACGTTATACGATG
P8	ACCACCTCTTAGCCTTAGCAC
P9	CGATGCTAGCTCACCGTTTGGACCTTGGGGCC
P10	CGTAGTCGACGATGGCGCCTCATCCCTGAAGC
P11	GCATGTACTAAGGAGGTTGTGTGAAACCAGTAACGTTATAC
P12	GTATAACGTTACTGGTTTCACACAACCTCCTTAGTACATGC
P13	ATCTAACACCGTGCGTGTTGGCTATTTTACCTCTGGCGGTG
P14	CACCGCCAGAGGTAAAATAGCCAACACGCACGGTGTTAGAT
P15	CCCTCGTGACCACCCTGGCCTACGCCCTGCAGTGCTTCGCCCG
P16	CGGGCGAAGCACTGCAGGGCGTAGGCCAGGGTGGTCACGAGGG
P17	CGCGGTATGGCATGATAGCGCCCGGAAGAGAGTCAATTCAGGGTGGTGAATTACGGCCCCAAGGTCCAAACG
P18	CAATCAACTGTTTACCTTGTGGAGCGACATCCAGAGGCACTTCACCGCTTGTTGGCTTCAGGGATGAGGCGCC
P19	CTGCGTCGACCGTTACCAATTATGACAACTTGAC
P20	CATTCGGCCGTTACTTGTACAGCTCGTCCATG
P21	TCAGCCTAGGGATTATGCTAGCAGGAGGAATTCACCATGGTG
P22	CAGTCCTAGGACTGAGCTAGCTATCAGCATAATTGGTAACGGTCGAC

Supplementary Table 2: Steady-state expression levels of CI and Cro in number of molecules at different temperatures.

T	XF204			XF214			XF224		
(°C)	CI*	Cro*	n (cells)	CI*	Cro*	n (cells)	CI*	Cro*	n (cells)
30	99 ± 4	11 ± 1	693	98 ± 3	13 ± 1	1232	95 ±3	1 ± 0.1	1367
31	89 ± 3	32 ± 4	1243	93 ± 3	17 ± 1	1461	90 ± 4	2 ± 0.2	762
32	90 ± 3	25 ± 2	1050	86 ± 3	14 ± 1	1392	79 ± 2	2 ± 0.3	1974
33	86 ± 4	60 ± 5	485	98 ± 6	28 ± 3	459	104 ± 6	3 ± 0.2	401
34	82 ± 5	275 ± 21	468	96 ± 6	40 ± 3	427	95 ± 5	2 ± 0.2	596
35	2 ± 1	846 ± 13	1080	24 ± 2	274 ± 6	1396	71 ± 3	3 ± 0.2	894
36	1 ± 1	918 ± 12	1384	7 ± 1	402 ± 4	1514	64 ± 2	6 ± 0.4	1493
36.5	4 ± 2	1112 ±16	1059	4 ± 1	489 ± 5	1493	54 ± 2	18 ± 1	1680
37	4 ± 4	1055 ± 24	512	1 ± 1	425 ± 7	618	14 ± 3	44 ± 5	176
38	N. D.	N. D.	N. D.	N. D.	N. D.	N. D.	10 ± 2	44 ± 3	266

*:All values were expressed as mean ± standard error.

Supplementary Table 3: smFISH probes used in the study. All the sequences were from 5’ to 3’.

LacI Probes	CI Probes	Cro Probes
tctgcgacatcgtataacgt	ggtttctttttgtgctcat	ttcagggttatgcgttgttc
ctgataagagacaccggcat	ctcaagctgctcttgtgtta	cccaaagcgcattgcataat
tggttcaccacgcgggaaac	aattgctttaaggcgacgtg	gagatctttagctgtcttgg
ttttcgcagaaacgtggctg	gggataagccaagttcattt	ttgttgatcgcgctttgata
cgcggttgggaatgtaattc	atcttgtctgcgacagattc	aaaatctttcggcctgcatg
ccaatcagcaacgactgttt	aataaagcaccaacgcctga	tccatcagcgtttatagtta
agatttaatcgccgcgacaa	gcatttaatgcattgatgcc	ttacctcttccgcataaacg
cttcgttctaccatcgacac	tttttgcaagcaatgcggcg	tttttgttactcgggaaggg
cgttgcgcgagaagattgtg	tcttcaacgctaactttgag	
gatagttaatgatcagccca	tctggcgattgaagggctaa	
taacgccggaacattagtgc	gcttcatacatctcgtagat	
atactgttgatgggtgtctg	aagtgacggctgcatactaa	
taccgtcttcatgggagaaa	cagggtactcatactcactt	
aatgcgaccagatgctccac	cctgcctgaacatgagaaaa	
ctaacagcgcgatttgctgg	tctaagctcaggtgagaaca	
gcgccgagacagaacttaat	ccgcatcacctttggtaaag	
gagatatttatgccagccag	gaatcactggctttttttggt	
cgctatcggctgaatttgat	ttcaacctcaagccagaatg	
tgaaaaccggacatggcact	ttggtgcggtcatggaatta	
attcagcatttgcatggttt	cattccgtcaggaaagcttg	
atctgatcgttggcaaccag	tgctcagggtcaacgagaat	
tatcccactaccgagatatc	gaaatcacctggctcaacag	
atgagctgtcttcggtatcg	taaactcatcacccccaagt	
gtggttaacggcgggatata	tccctgatcagtttcttgaa	
agcaggcgaaaatcctgttt	ttgtaaaaacacctgaccgc	
tgagagagttgcagcaagcg	attgggtactgtgggtttag	
agacgggcaacagctgattg	acaactctcattgcatggga	
agggtggtttttcttttcac	tgactagcgataactttccc	
gagaggcggtttgcgtattg	cagccaaacgtctcttcagg	
gccagctgcattaatgaatc		
ctttccagtcgggaaacctg		

Supplementary Table 4: Percentages of XF224 cells at different cell age thresholds showed similar four populations of [Cro, CI] mRNA expression measured using smFISH.

Cell age threshold	[L, L]	[H, L]	[L, H]	[H, H]
0.2	0.38	0.18	0.30	0.15
0.4	0.44	0.16	0.23	0.17
0.6	0.46	0.15	0.22	0.17
1	0.47	0.15	0.22	0.16
1*	0.42	0.19	0.26	0.12

*: Percentages of all XF224 cells showing four different populations by randomly scrambling the distribution of CI and Cro mRNA copy numbers in cells. There was a significant drop of [H, H] and {l, L] populations but increase of [H, L] and [L, H] populations, consistent with the expectation that [L, L] and [H, H] were unlikely caused by chance. s

Supplementary Table 5: Transition probability (per frame or 5 min) and time constants (min) from the four states in the column to the four states in the row, calculated from HMM-identified switching events in time traces of Cro and CI expression using the Master equation approach. The self-transition time constants are the dwell time of each states. These dwell times are consistent with what were directly determined by identified states using HMM (Table 1).

A: Transition probability using full-length time traces of all cells.

	[L, L]	[H, L]	[L, H]	[H, H]
[L, L]	0.703	0.260	0.025	0.013
[H, L]	0.016	0.860	0.003	0.121
[L, H]	0.061	0.022	0.818	0.098
[H, H]	0.008	0.015	0.080	0.897

B: Transition time constants (min) using full-length time traces of all cells.

	[L, L]	[H, L]	[L, H]	[H, H]
[L, L]	16	25	63	167
[H, L]	249	34	1660	41
[L, H]	75	828	26	41
[H, H]	2354	140	78	49

C: Transition probability using truncated time traces of young cells (age < 0.4).

	[L, L]	[H, L]	[L, H]	[H, H]
[L, L]	0.744	0.225	0.021	0.011
[H, L]	0.013	0.872	0.003	0.113
[L, H]	0.046	0.020	0.829	0.106
[H, H]	0.005	0.011	0.090	0.894

D: Transition time constants (min) using truncated time traces of young cells (age < 0.4).

	[L, L]	[H, L]	[L, H]	[H, H]
[L, L]	17	19	157	559
[H, L]	253	35	1859	42
[L, H]	72	1440	26	41
[H, H]	1961	284	54	44

Supplementary Table 6: Mean expression levels of CI and Cro and the corresponding dwell time of each state identified using HMM on truncated time traces of young cells (cell age < 0.4).

State [Cro, CI]	Cro* (molecules)	CI* (molecules)	n (frames)	Dwell Time* (min)	n§ (occurrence)
[L, L]	0.0 ± 0.01	0.0 ± 0.0	103	19 ± 2	28
[H, L]	3.8 ± 0.10	0.0 ± 0.0	689	36 ± 3	169
[L, H]	0.0 ± 0.0	3.9 ± 0.13	862	28 ± 1	269
[H, H]	4.5 ± 0.04	5.0 ± 0.05	1454	46 ± 3	391

*: Values were expressed as mean ± standard error.

§: Number of occurrence of each state in all time traces

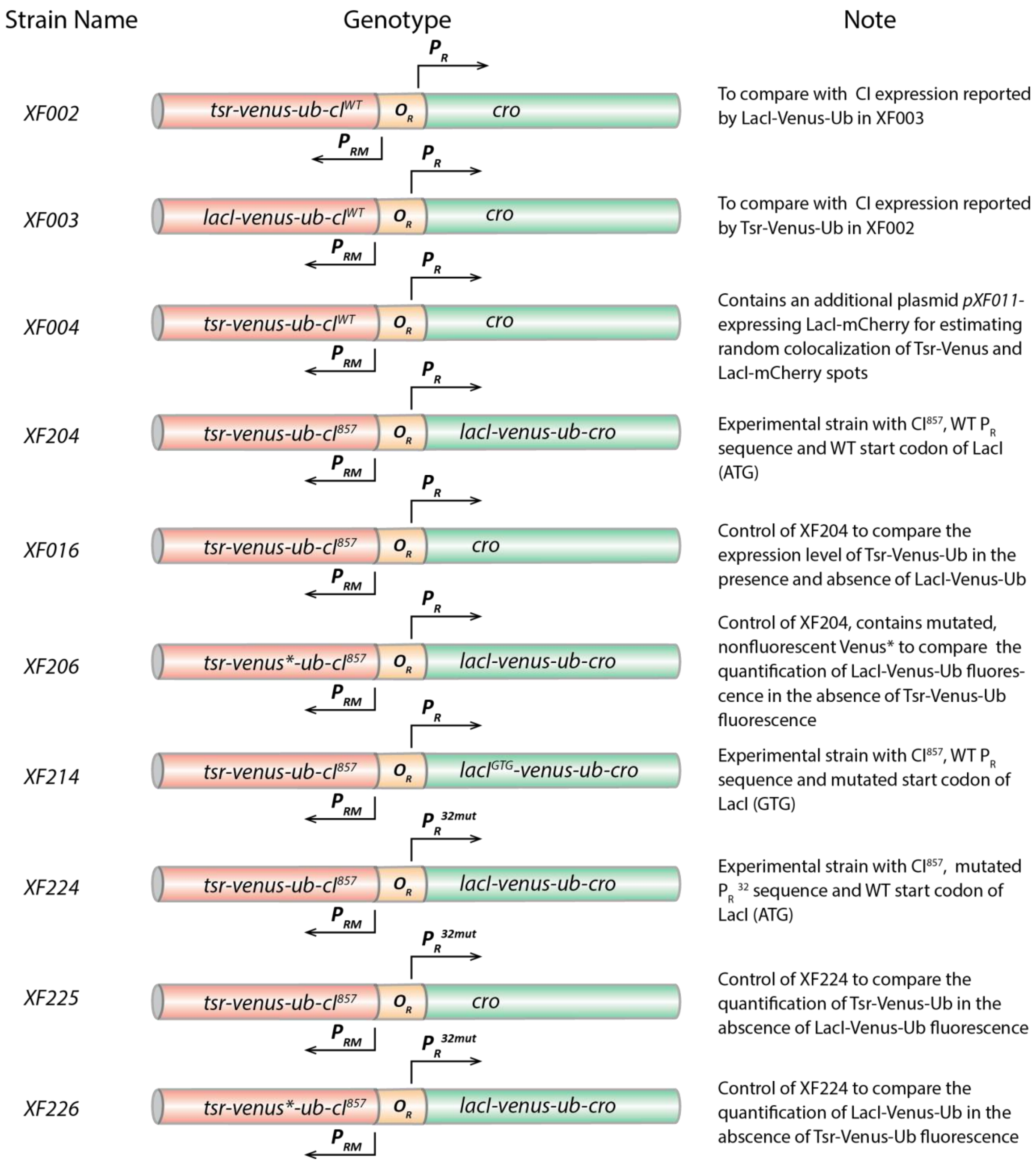

Supplementary Figure 1. Schematic drawing and experimental purpose of strains used in the study. All strains harbor the pZZ6 *lacO*^{256} plasmid for LacI-mCherry-Ub localization, and plasmid pCG001 (or its derivative) for expressing deubiquitinase UBP1. For exact genotypes please see Supplementary Table 1.

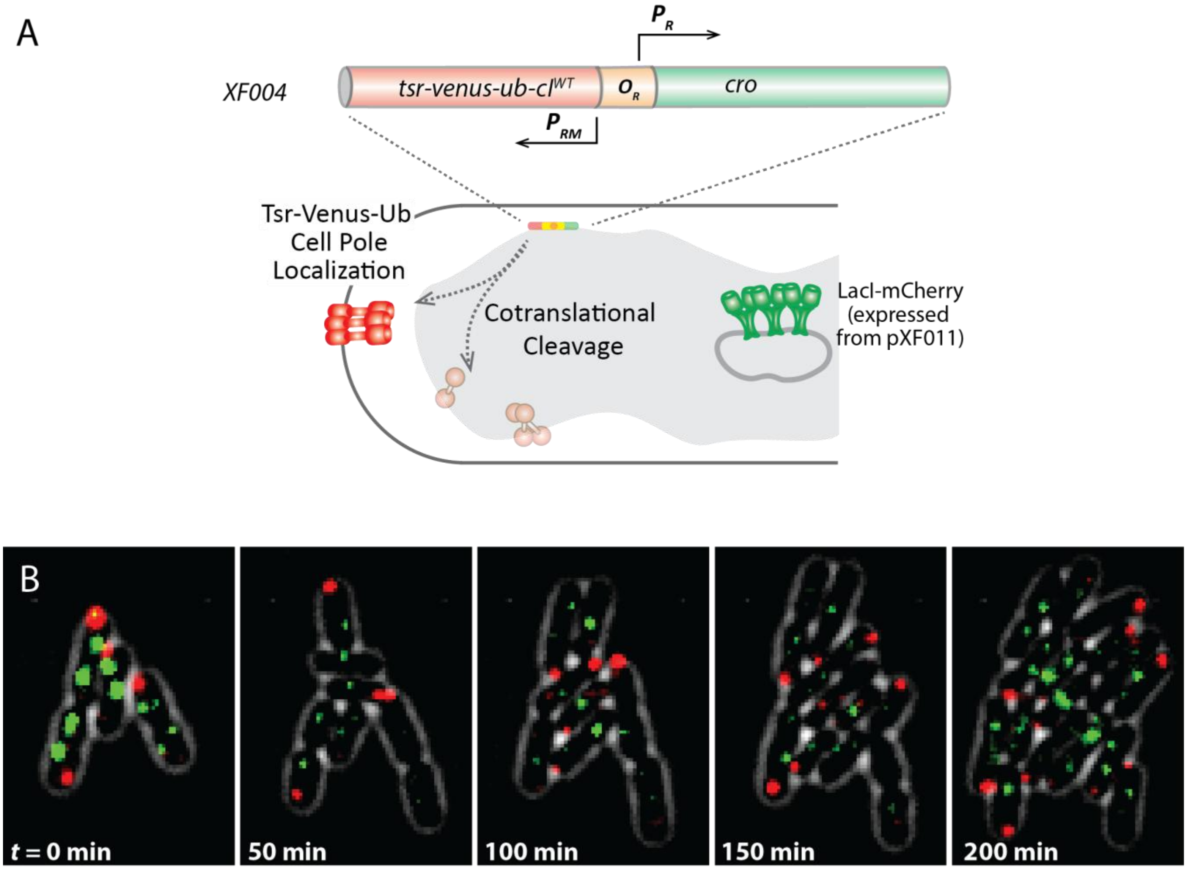

Supplementary Figure 2: Measurement of random colocalization between pole-localized Tsr-Venus (green) and midcell plasmid-localized LacI-mCherry (red) spots in strain XF004. (**A**) Schematic drawing of strain XF004, which expresses Tsr-Venus-Ub-CI^{Wt} from the chromosomal locus, and LacI-mCherry from an additional plasmid pXF011. The UBP1protease was constitutively expressed from pXF011 to separate Tsr-Venus-Ub from CI. LacI-mCherry localizes to the pZZ6 plasmid. (**B**) Representative montages from a time-lapse movie of XF004. Tsr-Venus-Ub spots were pseudo-colored red and LacI-mCherry spots green. In total 580 Tsr-Venus-Ub spots and 936 LacI-mCherry spots were counted, and among them 15 Tsr-Venus-Ub (2.6%) and LacI-mCherry spots (1.6%) colocalized by chance.

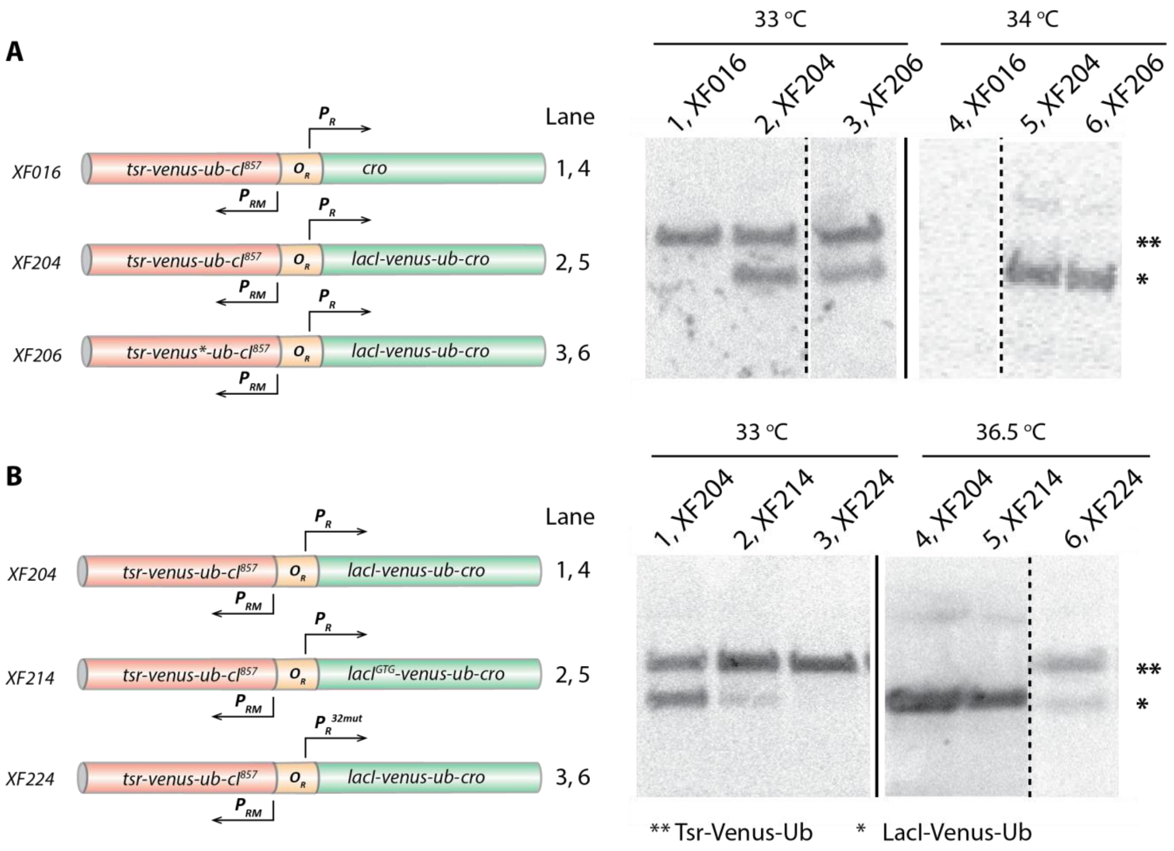

Supplementary Figure 3. Expression levels and switching behaviors of control strains harboring the UBP1 plasmid by Western Blotting using an anti-YFP antibody. (**A**) Tsr-Venus-Ub bands (**) indicative of CI expression were detected at similar levels in the presence of untagged Cro (Lane 1) or tagged LacI-Venus-Ub-Cro (Lane 2 and 3) at 33 °C. At this temperature, LacI-Venus-Ub bands (*) indicative of Cro expression were also detected in XF204 and XF206 (Lane 2 and 3). Tsr-Venu-Ub band disappeared in all the three strains at 34 °C (Lane 4, 5 and 6) while LacI-Venus-Ub bands were detected (Lane 5 and 6). Strain XF206 was identical to XF204 except that the Venus fluorophore contained two point mutations (G65A and G67A) to diminish its fluorescence to provide as a microscopy imaging control (Supplementary Table 1). These experiments demonstrated that the fusion of LacI-Venus-Ub and the subsequent cleavage from Cro did not change the

switching behavior of the switch at the two temperatures. (**B**) Comparison of switching behaviors of CI and Cro in three strains of different Cro expression levels (XF204, 214 and 224) at 33 °C and 36.5 °C. Tsr-Venus-Ub bands were detected in the three strains at 33°C, while LacI-Venus-Ub bands intensity decreased in the order of XF204>XF214>XF224 (Lane 1, 2 and 3). At 36.5 °C, Tsr-Venus-Ub bands disappeared in XF204 and XF214 (Lane 4 and 5), but remained faintly in strain XF224 (Lane 6), where Cro expression level was the lowest. Solid lines on Western blots indicated that the two adjacent images were from two different gels. Dashed lines indicated the two adjacent images were from the same gel but separated by other lanes. These experiments demonstrated that the proteolytic cleavages between Ub and CI or Cro were complete (no higher molecular weight bands of full length Tsr-Venus-Ub-CI or lacI-Venus-Ub-Cro) and that the three strains had different expression levels of Cro at same temperatures. Also see Supplementary Figure. 5.

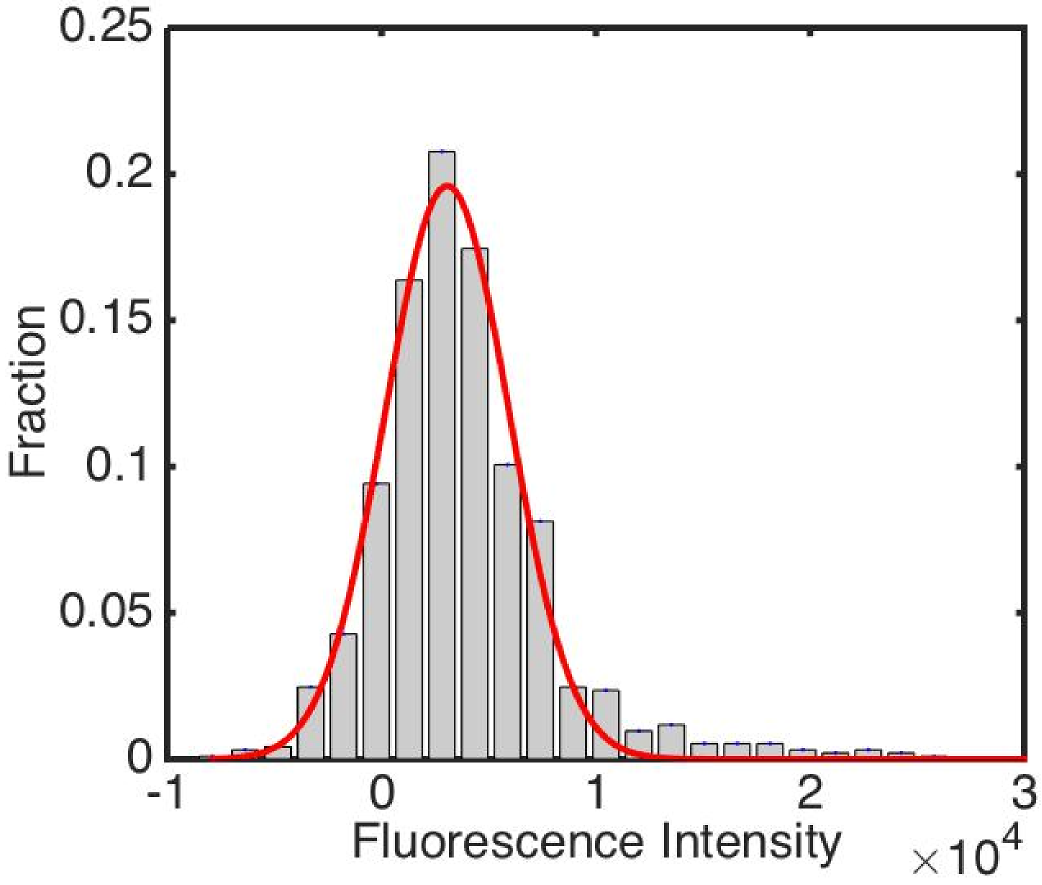

Supplementary Figure 4: Fluorescence intensity distribution of single Venus fluorophores (n = 934) after background subtraction. The distribution (gray) was fit with a single Gaussian function (red) with a peak value at 3064, which was used to convert integrated fluorescence intensity of Venus spots into the number of Venus molecules.

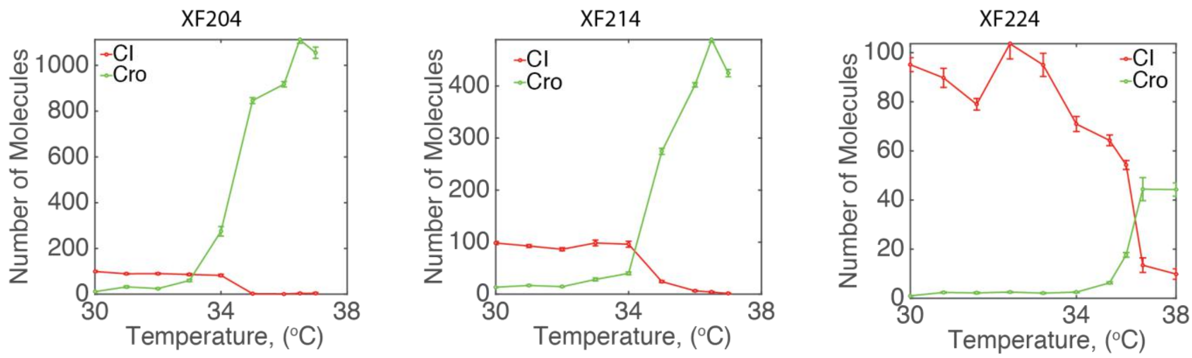

Supplementary Figure 5: Mean molecule numbers of CI (red) and Cro (green) of strains XF204, 214 and 224 at different temperatures. Note that in the three strains CI expression levels at low temperatures were comparable, while Cro expression levels were significantly different due to genetic mutations introduced to lower translation (XF214) or transcription (XF224) efficiency. Error bars are standard errors of the mean calculated by bootstrapping using custom Matlab codes. See Supplementary Table 2 for the corresponding data.

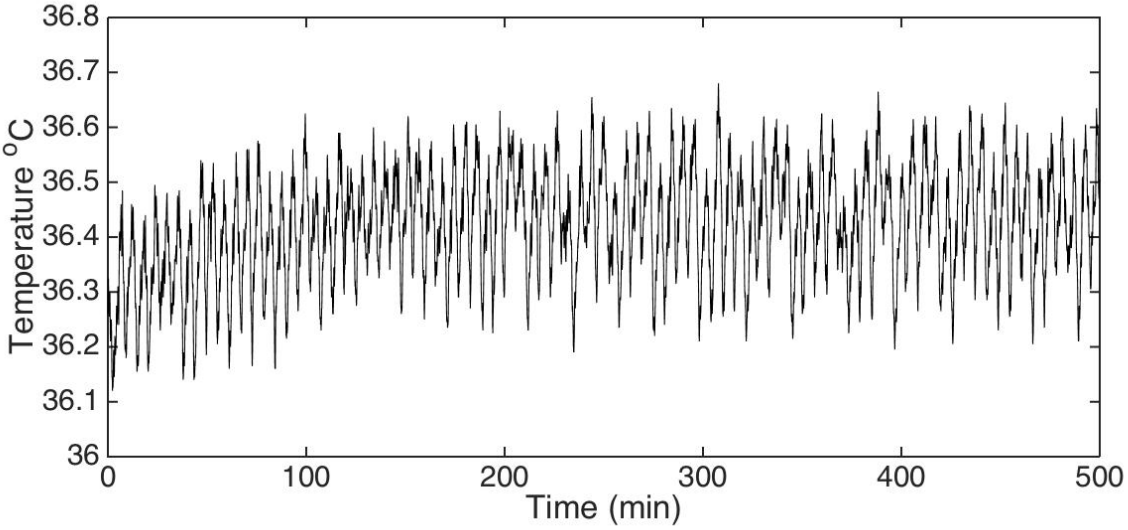

Supplementary Figure 6: A representative time trace of temperature fluctuations during a typical time-lapse experiment. In this trace the temperature was 36.4 ± 0.1 °C (μ ± s.d.).

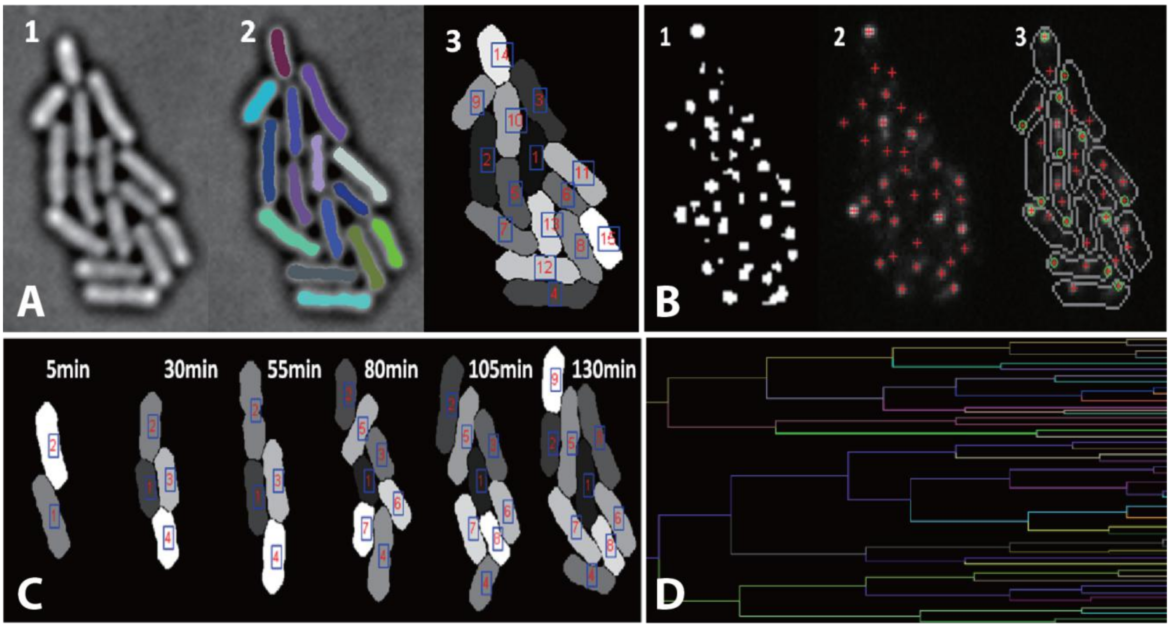

Supplementary Figure 7. Time-lapse movie processing and analysis procedure. (**A**) Bright-field cell images were segmented, aligned, and labeled using a custom MATLAB routine[6, 9]. (**B**) Fluorescent spots detection, quantification and attribution. (1) Fluorescent spots (white) above an intensity threshold were detected; (2) Each spot was fitted by a 2D Gaussian function to determine its total intensity and centroid position; (3) Each fluorescence spot was assigned to a cell based on cell outlines from (**A**) and attributed to either CI or Cro based on its position along the long axis of the cell. (**C**) Tracking cell lineages using the cell segmentation and labeling procedure in (**A**). (**D**) Two example lineage trees from the colony in (**C**).

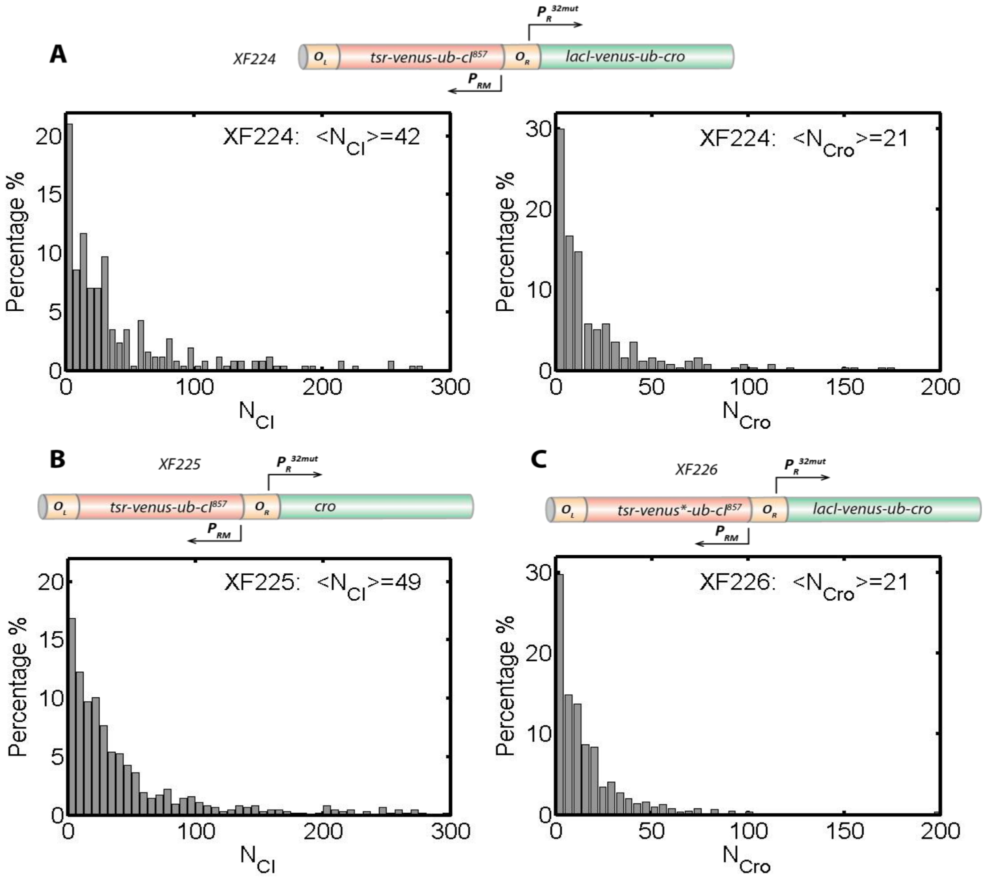

Supplementary Figure 8. Distributions of the number of CI and Cro molecules in XF224 (**A**) and the two control strains XF225 (**B**) and XF226 (**C**) measured at steady-state at 36.5 °C. Strains XF225 and XF226 harbored the same λ switch circuit, but with only CI or Cro labeled with a fluorescent localization tag (Supplementary Table 1). These two strains were used to verify that the copy numbers of CI and Cro identified in XF224 using distance thresholding were similar to that identified in XF225 or XF226 using total cellular fluorescence of Tsr-Venus-Ub or LacI-Venus-Cro only. For XF224, N_{CI} = 42 ± 3, N_{Cro} = 21 ± 2, μ ± s.e., n = 257 cells; for XF225, N_{CI} = 49 ± 3, μ ± s.e., n = 627 cells; for XF226, N_{Cro} = 21 ± 1, μ ± s.e., n = 632 cells. The similar distributions and mean expression levels of CI in XF224 and XF 225, and those of Cro in XF224 and XF226, demonstrated that the presence of both Tsr-Venus-Ub and LacI-Venus-Ub fluorescent tags in the same cells did not interfere with each other or prevent the accurate counting and identification of the corresponding fusion protein.

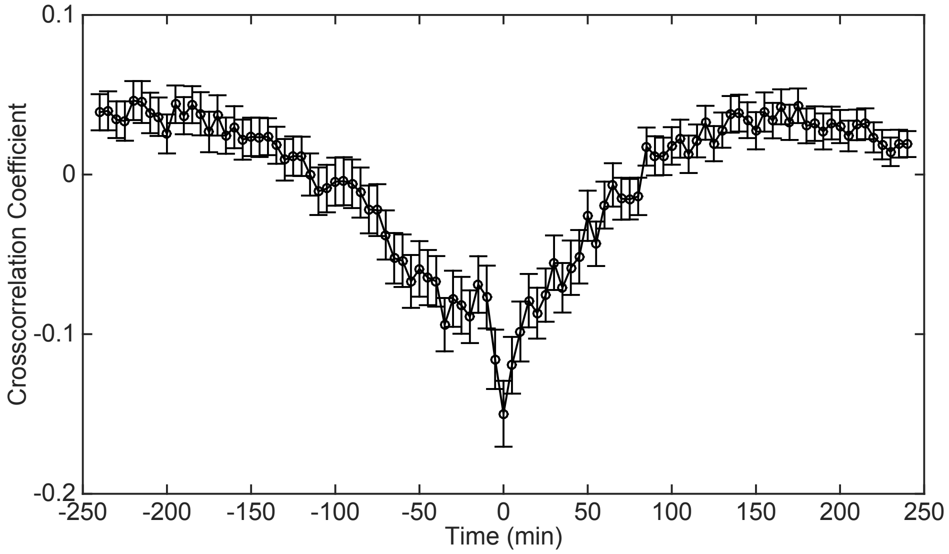

Supplementary Figure 9: Cross-correlation function of CI to Cro expression time traces. Cross correlation was calculated using the xcov function (unbiased, mean_subtracted) in Matlab.

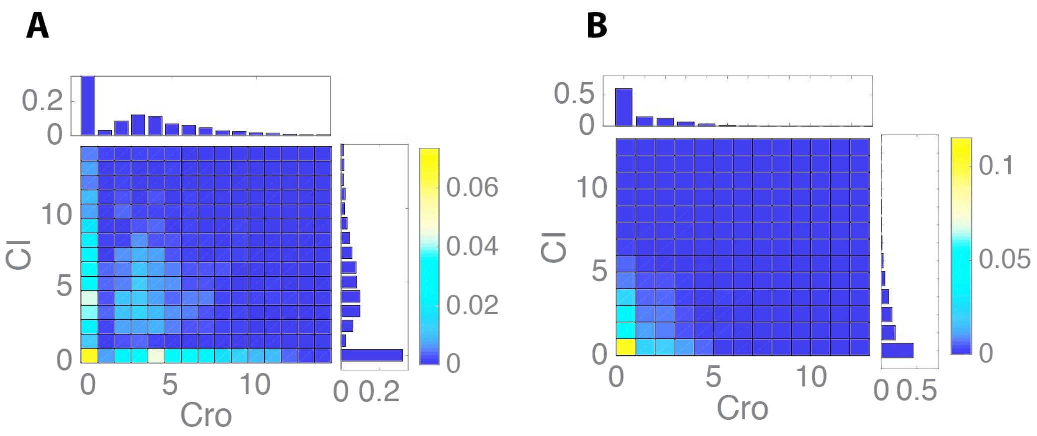

Supplementary Figure 10: 2D histograms of CI and Cro protein numbers (**A**) measured in time-lapse movies using young cells (cell age <= 0.4) and CI and Cro mRNA numbers measured in smFISH using young small cells with cell area <= 0.4 of total population (**B**). In these young cells the two additional populations of expressing CI and Cro both, or neither CI nor Cro, were still clearly visible. Color bars and y-axes of the 1D histograms were fractions of cells.

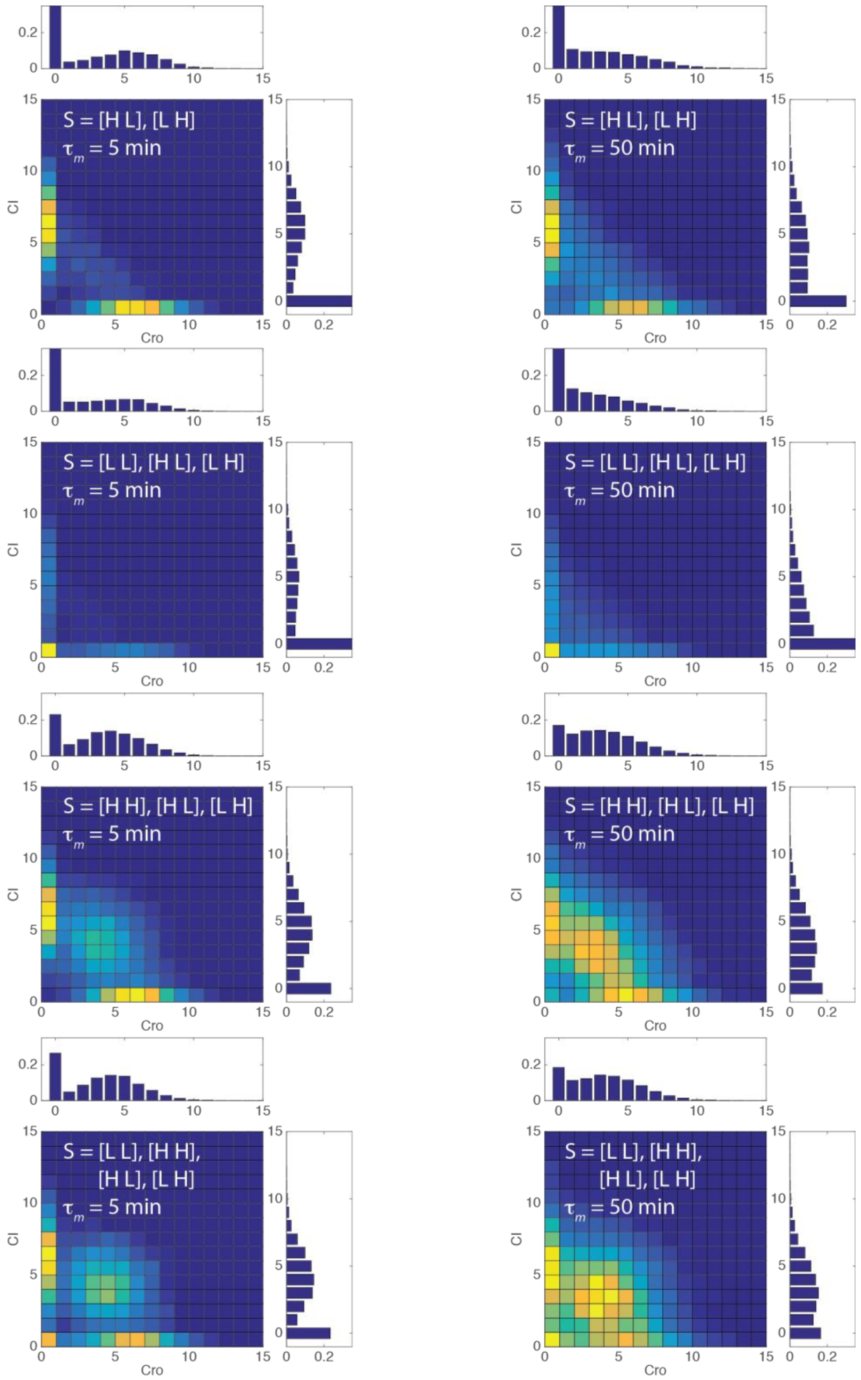

Supplementary Figure 11: 2D histograms of CI and Cro expression numbers using simulations of varying population numbers and maturation time. The corresponding 1D histograms of Cro and CI of each condition were plotted at the top and side of the 2D histogram respectively. In all the conditions simulated, longer maturation time τ_m only increased the spread of each population but did not add new populations.

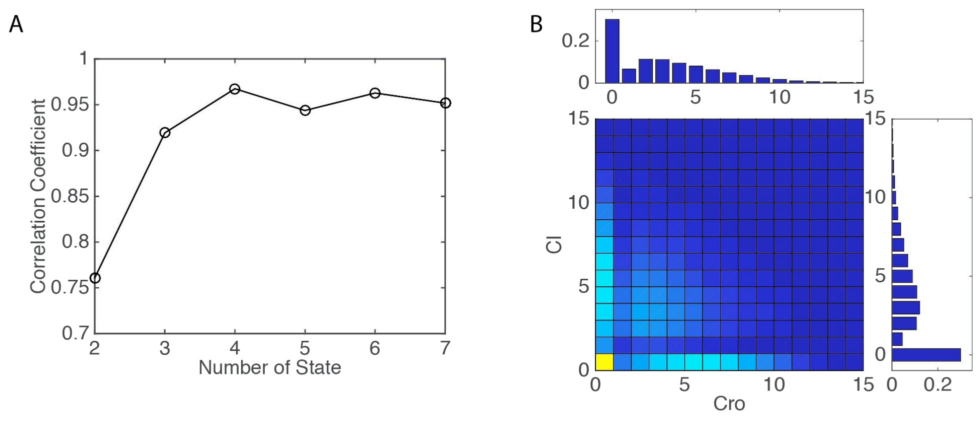

Supplementary Figure 12. HMM data analysis of XF224 time traces. (**A**) Correlation coefficients between the HMM-simulated 2D histogram at different number of states and the experimentally measured 2D histogram of CI and Cro expression per 5-min frame in time traces. (**B**) Simulated 2D histogram of CI and Cro expression per 5-min frame using a 4-state HMM.

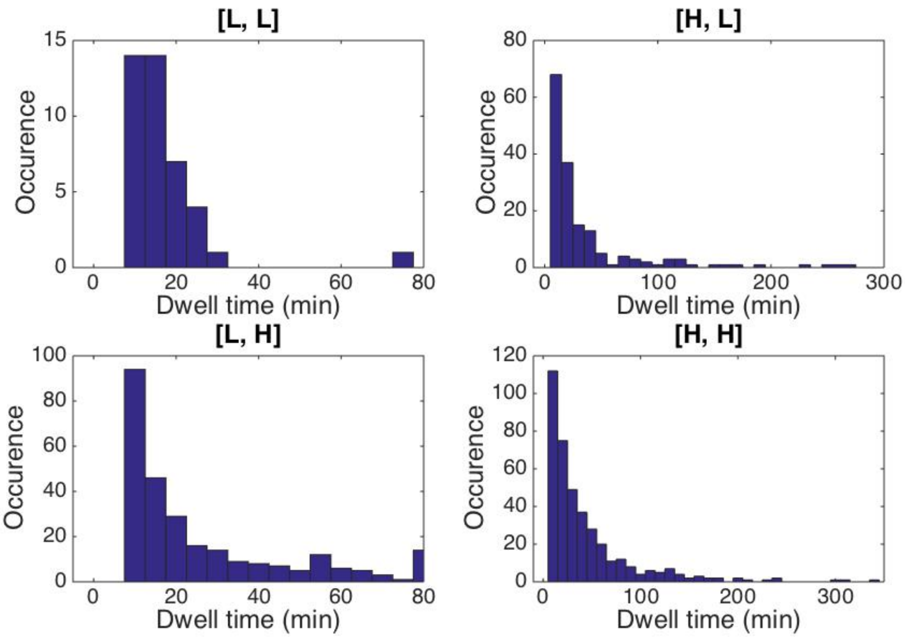

Supplementary Figure 13: Dwell time distribution of four expression states identified using HMM. The mean dwell time for each state was summarized in Table 1.

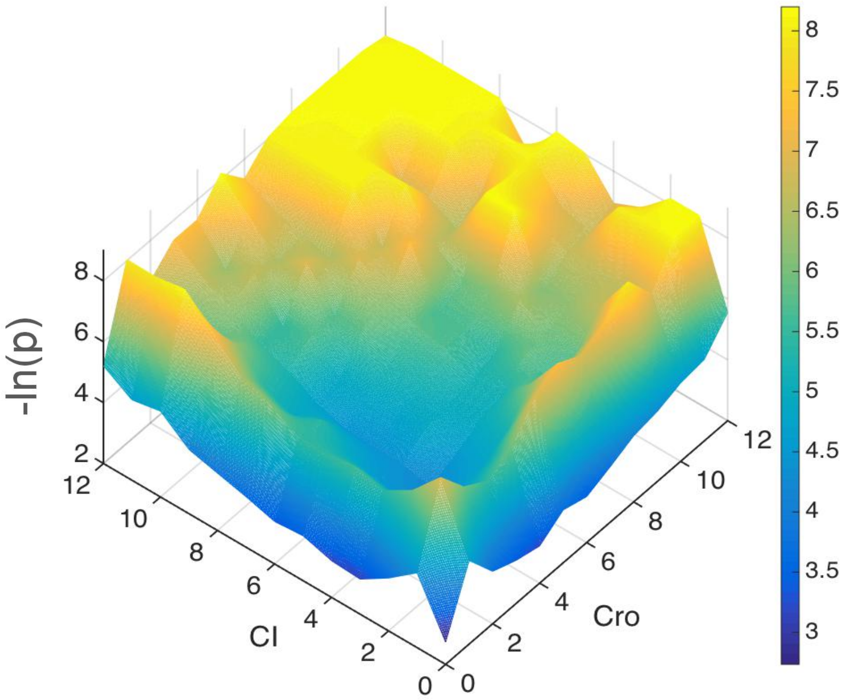

Supplementary Figure 14: Potential landscape of CI and Cro expression levels every 5-min calculated using truncated time traces of young cells (cell age < 0.4).

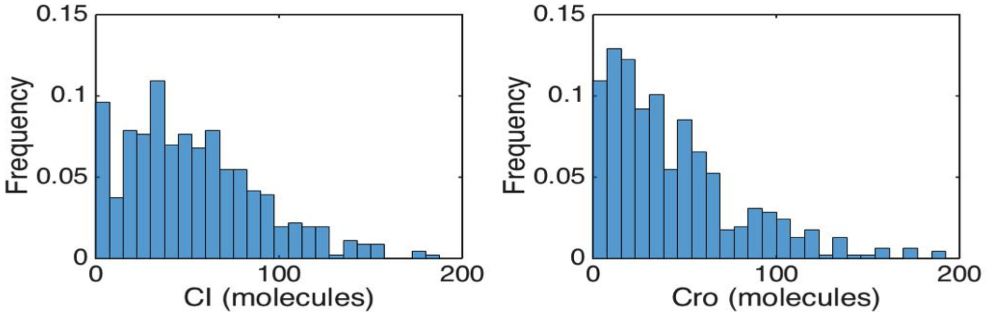

Supplementary Figure 15: Histograms of number of CI (**A**) and Cro (**B**) molecules produced in each cell cycle. The mean expression level was 53 ± 37 molecules, μ ± s.d, for CI, and 44 ± 37 molecules for Cro, n = 457 cell cycles.

Supplementary Note 1: Quantification of potential landscape

The 2D histogram of CI and Cro expression per 5-min frame constructed using time traces (Figure 3 in the main text) was also the probability landscape *p* with respect to CI and Cro. The potential landscape U is closely related to the probability landscape by the relationship U = -ln(*p*) [10] From the quantified potential landscape, we identified four basins of attractions (the location of the basin is defined as the local mimimum point on the potential landscape). The probability landscape and potential landscape give global descriptions of the functional states or cell fates by quantifying their underlying weights.

Supplementary Note 2: Maximum likelihood estimate of HMM parameters

A maximum likelihood estimate[11] of HMM parameters was performed globally on all the time traces. Specifically, we evaluated the likelihood function for each time trace, and then found the overall global fitting parameters that gave the best performance of all the traces in terms of their likelihood functions. Multiple random initial parameters (number of states and transition probabilities) were used to start the iterative HMM analysis and to ensure convergence to the global minimum. The Baum–Welch algorithm [11] was used to re-estimate the parameters at the end of each iteration. Steady-state condition was enforced on the re-estimated parameters in each iteration. The condition can be described as $\sum i \neq j\ \pi_i \bullet a_{ij} = \sum j \neq i\ \pi_j \bullet a_{ji}$, where $a_{i,j}$ is the transition probability from state *i* to state *j* per time bin, and π_i is the probability of state *i* in steady state.

HMM analysis does not directly provide an estimate for state number N (ref[11]). Therefore, we performed HMM analysis with different values of N, from 2 to 7, and calculated the correlation of the resulting 2D histogram of CI and Cro expression numbers with that obtained from

experimental time traces. We chose N = 4 as the best number of states because the correlation coefficient (~ 0.9) was the highest when state number increased to 4, and did not further increase significantly after N > 4 (Supplementary Figure. 12A). The corresponding 2D histogram of CI and Cro expression numbers with N = 4 in HMM was highly similar to that obtained experimentally (Supplementary Figure. 12B). The corresponding transition probability between each state was summarized in Supplementary Table 5.

Supplementary Note 3: Quantify transition time constants using the master equation approach

Transition probabilities identified from HMM analysis are closely correlated but not directly linked to transition rates, because there are infinite numbers of kinetic paths to transition between two states, and the rate is the weighted average of these paths. To identify transition rates between two states we used the master equation approach. The master equation can be written as

$$\frac{d}{dt}\begin{pmatrix} P_1 \\ P_2 \\ P_3 \\ P_4 \end{pmatrix} = \begin{bmatrix} k_{11} & k_{12} & k_{13} & k_{14} \\ k_{21} & k_{22} & k_{23} & k_{24} \\ k_{31} & k_{32} & k_{33} & k_{34} \\ k_{41} & k_{42} & k_{43} & k_{44} \end{bmatrix}\begin{pmatrix} P_1 \\ P_2 \\ P_3 \\ P_4 \end{pmatrix} \qquad (1)$$

where P_1, P_2, P_3, and P_4 are the probabilities of [L,L], [H,L], [L,H], [H,H] states, respectively, k_{ij} (i,j=1,2,3,4) is the transition rate from P_j to P_i. The simple kinetic model can be shown in the following reaction scheme.

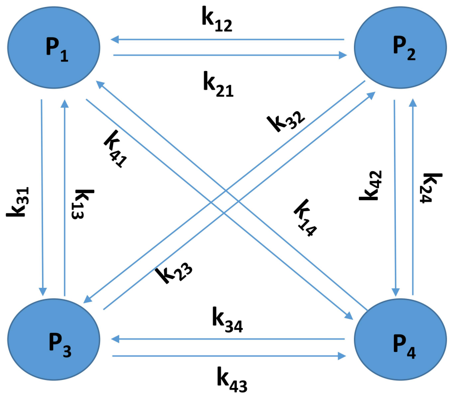

From this reaction scheme, the transition rate k_{11} is the rate at which P_1 decreases. Due to the probability conservation, $k_{11}= -(k_{21}+k_{31}+k_{41})$. Similar results can be obtained for the other states:

$k_{22} = -(k_{12}+k_{32}+k_{42})$,

$k_{33} = -(k_{13}+k_{23}+k_{43})$,

$k_{44} = -(k_{14}+k_{24}+k_{34})$

The master equation can then be written as follows:

$$\frac{d}{dt}\begin{pmatrix} P_1 \\ P_2 \\ P_3 \\ P_4 \end{pmatrix} = \begin{bmatrix} -(k_{21}+k_{31}+k_{41}) & k_{12} & k_{13} & k_{14} \\ k_{21} & -(k_{12}+k_{32}+k_{42}) & k_{23} & k_{24} \\ k_{31} & k_{32} & -(k_{13}+k_{23}+k_{43}) & k_{34} \\ k_{41} & k_{42} & k_{43} & -(k_{14}+k_{24}+k_{34}) \end{bmatrix} \begin{pmatrix} P_1 \\ P_2 \\ P_3 \\ P_4 \end{pmatrix}$$

Once we know the numerical values of the rate matrix of the master equation, the time scales of switching can be directly quantified. From the experimental time traces, we can use HMM to identify the underlying states and quantify the corresponding transition probability matrix. Our goal is thus to infer the rate matrix in the master equation from the transition probability matrix of HMM based on the experimental data. The connection between the rate matrix in the master equation and probability matrix in the HMM can be established as follows. Through the numerical method, we can obtain the probability of 5 minutes (observation time window) later from the

master equation. This probability corresponds to an element of the transition probability matrix, for example, when P_1 =1, while the others are all zero. The calculations of the four probabilities of 5 minutes later give the transition probability from P_1 to P_j (j=1,2,3,4), respectively. Therefore, for a given set of parameters, the corresponding transition probability matrix can be obtained by using different initial values. In this way, we can establish the connections between the rate matrix in master equation and probability matrix of HMM. Using Genetic Algorithm[12], we can find the optimal set of parameters, so that the numerical values of the transition rate matrix in the master equation can be inferred faithfully from the transition probability matrix obtained by HMM based on the experimental data. The transition time constants were calculated as the reciprocal of respective rates in the master equation and summarized in Supplementary Table 5. The diagonal elements of the transition time matrix correspond to the average resident times for the associated states from the master equation. The dwell time is the average residence time of a state directly from the experimental time traces once each state is identified by HMM.

Supplementary Note 4: Cell-age dependence analysis

Based on the concern that the four states may be caused by the independent expressions of CI and Cro from two copies of genes due to chromosome replication, we extracted young cells data from all time traces. For each cell cycle we defined cell age of 0 at the first frame and 1 at the last frame, and calculated cell age at each frame in between by normalizing the time of that frame to the total number of frames in the same cell cycle. We then selected all the frames with cell age < 0.4 in each cell cycle as young cell data, and plotted the corresponding 2D histogram (Supplementary Figure. 10A). The 2D histogram of young cells still showed clear four populations similar to that from all cell data (Fig. 3E). Consistent with this observation, potential landscape (Supplementary

Figure. 14) and four-state HMM analysis of young cell data showed similar transition probability and time constants (Supplementary Table 5).

For smFISH data, we calculated cell age based on cell size. Single cells were segmented and cell area measured using a custom Matlab code. A cell *i*'s age a_i was calculated by the equation $a_i = \frac{S_i - \min(S)}{\max(S) - \min(S)}$, where S is the measured cell area of cell i, min(S) was the minimal cell area of the population, and max(S) was the maximal cell area of the same population. The 2D histogram of CI and Cro mRNA numbers using cells with age ≤ 0.4 were plotted in Supplementary Figure. 10B. The four populations of CI and Cro mRNA expression levels were defined using a threshold of mRNA copy number = 1, such that a cell with no CI or Cro mRNA molecules belonged to the [L, L] population, and a cell with one or more molecules of CI and Cro mRNAs belonged to the [H, H] population and so forth. The percentages of cells showing four different populations of mRNA expression levels using different cell age threshold did not exhibit significant difference (Supplementary Table 4), demonstrating that there was little cell-age dependence of the four populations.

Supplementary Note 5: Fluorophore maturation analysis

The Venus fluorophore has the fastest maturation rate among all known fluorescent proteins (~ 4 to 7 min in live *E. coli* cells[3,6], therefore we did not expect that the stochastic maturation of individual Venus fluorophores would cause substantial temporal overlaps between adjacent frames (five minuets intervals) to contribute significantly to the presence of the [H, H] population. Nevertheless, we performed simulations to verify the possibility. We used the transition probability matrix to generate a series of state trajectories using HMM. At a given time point, depending on the state ([L,L], [L,H], [H,L] or [H,H]) it was in, a number of Venus molecules

representative of CI or Cro was generated. Each molecule was then assigned a maturation probability after 5 min using a maturation time constant that followed an exponential distribution. The maturation probability of a Venus molecule was then compared with a random number between 0 and 1. If the maturation probability was greater than the random number, the molecule was considered mature and would be detected in this frame. If not, its maturation probability will be recalculated in the next five minutes. The process was repeated for 500 min to generate a production time trace of fluorescent Venus molecules. A total of 100 time traces were simulated using different number of states and different mutation time constants, and the resulting 2D histogram of CI and Cro were plotted in Supplementary Figure. 11. In all the conditions simulated, longer maturation time τ_m only increased the spread of each population but did not add new populations.

References

1. Brown, S., Ferm, J., Woody, S., Gussin, G., Selection for mutations in the PR promoter of bacteriophage lambda. *Nucleic Acids Res* **18**, 5961-5967 (1990).
2. Kuhlman, T. E., Cox, E. C., Site-specific chromosomal integration of large synthetic constructs. *Nucleic Acids Res* **38**, e92 (2010).
3. Yu, J., Xiao, J., Ren, X., Lao, K., Xie, X. S., Probing gene expression in live cells, one protein molecule at a time. *Science.* **311**, 1600-1603. (2006).
4. Tobias, J. W., Varshavsky, A., Cloning and functional analysis of the ubiquitin-specific protease gene UBP1 of Saccharomyces cerevisiae. *J Biol Chem* **266**, 12021-12028. (1991).
5. Pogliano, J., Ho, T. Q., Zhong, Z., Helinski, D. R., Multicopy plasmids are clustered and localized in Escherichia coli. *Proc Natl Acad Sci U S A* **98**, 4486-4491 (2001).
6. Hensel, Z. *et al.*, Stochastic expression dynamics of a transcription factor revealed by single-molecule noise analysis. *Nat Struct Mol Biol* **19**, 797-802 (2012).
7. Hensel, Z., Weng, X., Lagda, A. C., Xiao, J**.**, Transcription-factor-mediated DNA looping probed by high-resolution, single-molecule imaging in live E. coli cells. *PLoS Biol* **11**, e1001591 (2013).
8. Anderson, J., in *iGEM2006_Berkeley*. (http://parts.igem.org/Part:BBa_J23103, 2006).
9. Hensel, Z., Fang, X., Xiao, J., Single-molecule imaging of gene regulation in vivo using cotranslational activation by Cleavage (CoTrAC). *Journal of visualized experiments : JoVE*, e50042 (2013).
10. Wang, J., Xu, L. & Wang, E. Potential landscape and flux framework of nonequilibrium networks: robustness, dissipation, and coherence of biochemical oscillations. *Proc Natl Acad Sci U S A* **105**, 12271-12276 (2008).(10)

11 Baum, L. E. & Petrie, T. Statistical Inference for Probabilistic Functions of Finite State Markov Chains. *The Annals of Mathematical Statistics*, 1554-1563, doi:10.1214/aoms/1177699147 (1966).(11)

12 Mitchell, M. *An introduction to genetic algorithms*. (MIT press, 1998).(12)
