# Supplementary material for: Cell fate potentials and switching kinetics uncovered in a classic bistable genetic switch
Source: Nat Commun. 2018 Jul 17;9:2787. doi: 10.1038/s41467-018-05071-1 (PMC6050291; doi:10.1038/s41467-018-05071-1)
Supplement: Supplementary file 3 — Description of Additional Supplementary Files [file 41467_2018_5071_MOESM3_ESM.pdf]

Description of Additional Supplementary Files

File Name: Supplementary Movie 1
Description: Time-lapse movie of an XF004 colony expressing Tsr-Venus-Ub-CI (green) integrated in the chromosome and LacI-mCherry (red) from a plasmid. Movie was taken using the same imaging condition as that for XF224 time-lapse movies.

File Name: Supplementary Movie 2
Description: Time-lapse movie of an XF002 colony expressing Tsr-Venus-Ub-CI (yellow) only. Imaging condition was the same as that for XF224 time-lapse movies.

File Name: Supplementary Movie 3
Description: Time-lapse movie of an XF003 colony expressing LacI-Venus-Ub-CI (yellow) only. Imaging condition was the same as that for XF224 time-lapse movies.

File Name: Supplementary Movie 4
Description: Time-lapse movies of two XF224 colonies expressing Tsr-Venus-UbCI857 and LacI-Venus-Ub-Cro. Imaging condition was described in Material and Methods. Frame rate was one per 5 minutes.

File Name: Supplementary Movie 5
Description: Time-lapse movies of two XF224 colonies expressing Tsr-Venus-UbCI857 and LacI-Venus-Ub-Cro. Imaging condition was described in Material and Methods. Frame rate was one per 5 minutes.
